# Optical Coherence Tomographic Findings in Berlin’s Edema

**Published:** 2010-04

**Authors:** Leila El Matri, Ahmed Chebil, Fedra Kort, Rym Bouraoui, Leila Largueche, Fatma Mghaieth

**Affiliations:** Department of Ophthalmology, Hedi Rais Institute of Ophthalmology, Tunis, Tunisia

**Keywords:** Berlin’s Edema, Macular Edema, OCT, Ocular Trauma

## Abstract

**Purpose:**

To describe optical coherence tomography (OCT) findings in a patient with Berlin’s edema following blunt ocular trauma.

**Case Report:**

A 26-year-old man presented with acute loss of vision in his left eye following blunt trauma. He underwent a complete ophthalmologic examination and OCT. Fundus examination revealed abnormal yellow discoloration in the macula. OCT disclosed thickening of outer retinal structures and increased reflectivity in the area of photoreceptor outer segments with preservation of inner retinal architecture. Re-examination was conducted one month later at the time which OCT changes resolved leading to a surprisingly normal appearance.

**Conclusion:**

OCT can be a useful tool in the diagnosis and follow-up of eyes with Berlin’s edema and may reveal ultrastructural macular changes.

## INTRODUCTION

Optical coherence tomography (OCT) is of great value in the management of patients with posterior segment trauma. It defines anatomic details to confirm the diagnosis and may allow for a better understanding of the pathogenesis, especially in acute traumatic maculopathy.^1^ Herein, we report OCT findings in the diagnosis and follow-up of Berlin’s edema in a patient with acute traumatic maculopathy and no other apparent ocular injury.

## CASE REPORT

**Figure 1:**
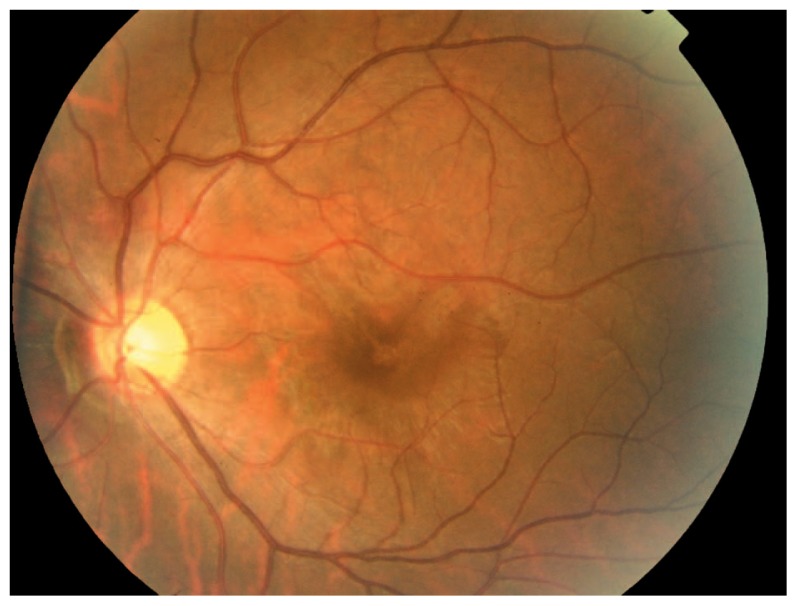
Fundus examination revealed abnormal yellow discoloration of the macular area with juxtapapillary subretinal haemorrhage.

**Figure 2:**
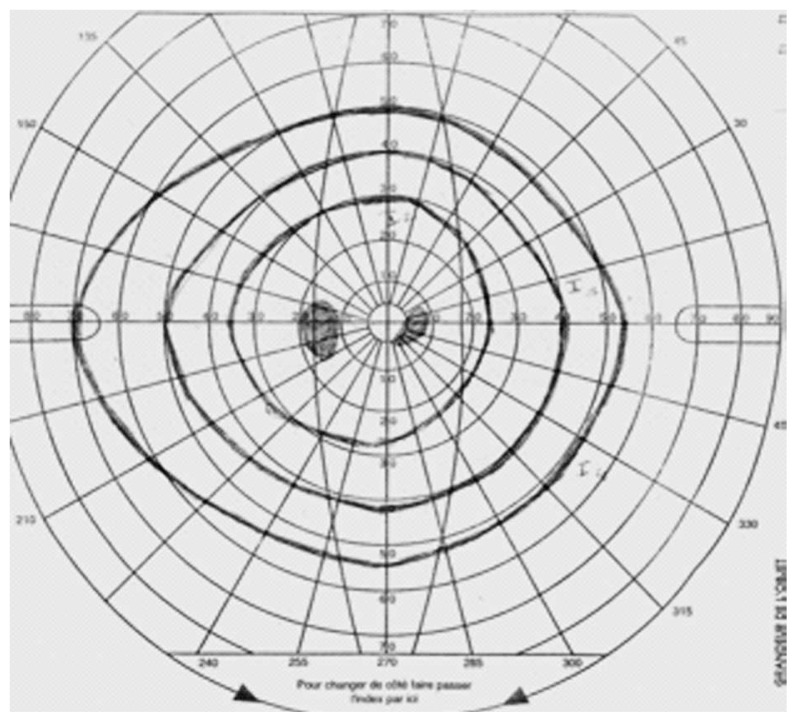
Goldmann perimetry disclosed an absolute central scotoma.

**Figure 3:**
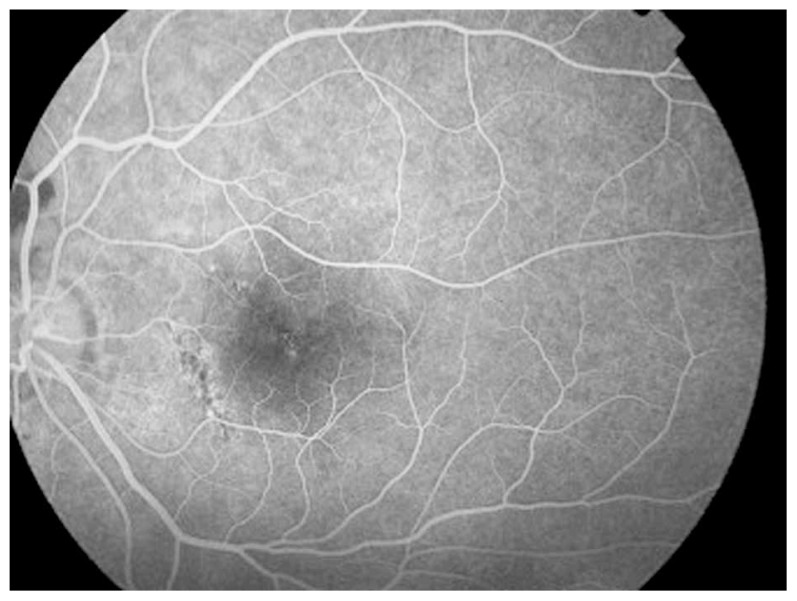
Fluorescein angiography revealed blocked fluorescence in the macula.

**Figure 4:**
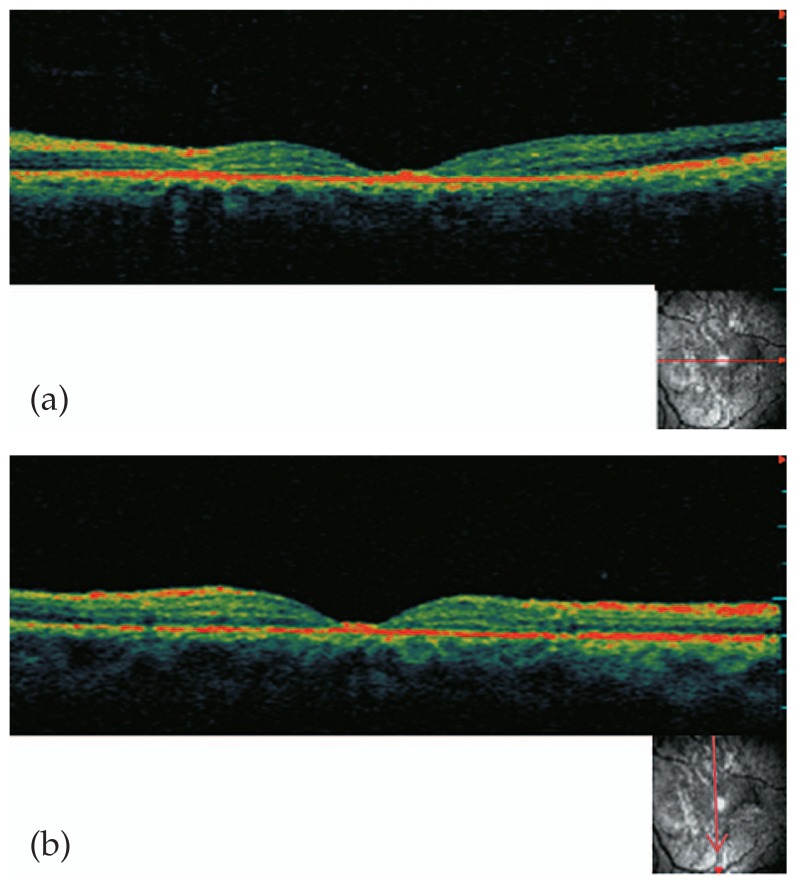
**(a)** Vertical and **(b)** horizontal linear optical coherence tomography scans upon presentation revealed thickening of outer retinal structures and increased reflectivity in the area of the photoreceptor outer segments with preservation of inner retinal architecture; central foveal thickness was 115 μm.

**Figure 5:**
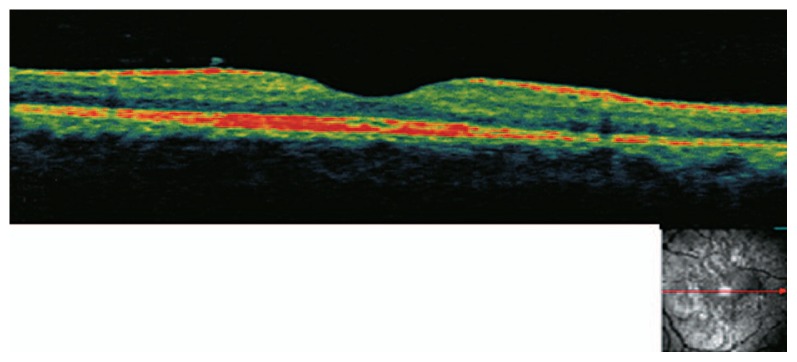
Optical coherence tomography one month later shows regression of foveal thinning with hyperreflectivity in the area of the photoreceptor outer segments, central foveal thickness was increased to 231 μm.

A 26-year-old man presented with acute loss of vision in his left eye following blunt trauma. Best-corrected visual acuity (BCVA) was 20/20 and 20/200 in the right and left eyes, respectively. Examination of the left eye showed a normal anterior segment. Fundus examination revealed abnormal yellow discoloration in the macular area with juxtapapillary subretinal hemorrhage, but the optic disc appeared normal (Fig. 1). All examinations of the right eye were normal. Goldmann perimetry revealed an absolute central scotoma (Fig. 2). Fluorescein angiography revealed blocked fluorescence in the macula (Fig. 3). Vertical and horizontal linear OCT Scans (OCT/SLO, OTI, Toronto, Ontario, Canada) revealed thickening of outer retinal structures and increased reflectivity in the area of the photoreceptor outer segments with preservation of inner retinal architecture; central foveal thickness was 115 μm (Fig. 4). One month later, BCVA of the left eye improved to 20/20. There was no evidence of significant scarring on ophthalmoscopy. Repeat OCT showed a surprisingly normal pattern with central foveal thickness of 231 μm (Fig. 5).

## DISCUSSION

Several characteristics make OCT an effective tool for management of patients with posterior segment trauma. OCT is more comfortable for the traumatized patient than other imaging techniques because it is a non-contact method utilizing infrared illumination. OCT is also highly sensitive in identifying subtle anatomic changes.^2^ Berlin’s edema is an acute traumatic maculopathy characterized by retinal opacification. Histopathologic studies have shown that it is characterized by disruption of photoreceptor outer segments and retinal pigment epithelial damage.^3^ The pathogenesis of Berlin’s edema is uncertain, but Pulido et al^4^ reported that breakdown of the blood-retinal barrier is one possible factor.

The mechanism of injury is presumed to be blunt force transmitted to the retina due to rapid deceleration of ocular tissues. Liem et al^5^ suggested that retinal opacification in Berlin’s edema is accompanied by a traumatic lesion at the level of the photoreceptor-retinal pigment epithelium complex. This is supported by histopathologic data on commotio retinae, which revealed moderate photoreceptor outer segment disruption and retinal pigment epithelial damage implying that such damage might be reversible. The severity and extent of involvement vary with the severity of trauma.

In the patient described herein, OCT showed involvement of the outer photoreceptor segment zone with preservation of inner retinal architecture. The major site of retinal damage seems to be at the level of the photoreceptor outer segments, which correlates with previously described histopathology.^3^ Increased reflectivity on OCT in the acute phase probably represents photoreceptor outer segment disruption, which is reversible depending on the extent of the initial trauma. Pham et al^1^ revealed neurosensory retinal detachment of the fovea in a patient with acute traumatic maculopathy.

OCT changes resolved approximately one month after the trauma in our patient accompanied by complete visual recovery. In addition to its diagnostic interest, OCT may also have a prognostic value. If the disruption involves only the photoreceptor outer segments, functional recovery is possible as was observed in our case. However, if the trauma is more severe, the inner segments of the photoreceptors may be damaged with the risk of cellular necrosis and consequent atrophic changes in the fovea.^3^ In summary, OCT can be a useful tool in eyes with Berlin’s edema, it may reveal persisting macular changes and explain poor visual recovery. It is also useful in delineating ultrastructural changes in the fovea following trauma.
